# Focal MRI and Learning Disability with Reduced Automaticity

**DOI:** 10.15844/pedneurbriefs-29-9-4

**Published:** 2015-09

**Authors:** J. Gordon Millichap

**Affiliations:** 1 Division of Neurology, Ann & Robert H. Lurie Children’s Hospital of Chicago, Chicago, IL; 2 Departments of Pediatrics and Neurology, Northwestern University Feinberg School of Medicine, Chicago, IL

**Keywords:** Automaticity, Brain MRI, Learning Disability

## Abstract

Investigators from the Boston Children’s Hospital, Harvard Medical School, MA, performed a retrospective analysis of 1,587 children referred for a learning disability, and 127 had a focal deficit demonstrated on either a neurologic or neuropsychological evaluation.

Investigators from the Boston Children’s Hospital, Harvard Medical School, MA, performed a retrospective analysis of 1,587 children referred for a learning disability, and 127 had a focal deficit demonstrated on either a neurologic or neuropsychological evaluation. Children with abnormalities in learning and a focal deficit were compared in terms of deficits in automaticity or difficulty in changing from controlled to automatic processing. Automaticity was measured by a Rapid Automatized Naming, Rapid Alternating Stimulus Naming, or the timed motor performance battery from the Physical and Neurological Examination for Soft Signs. Data were compared in children with and without disorders of automaticity regarding type of brain structure abnormality. Of 87 with a brain MRI, 40 (46%) were abnormal; abnormalities involved cerebellum (5), white matter (25), corpus callosum (9), and gray matter (11). These with focal abnormalities were compared with a sample of 150 patients with learning disabilities and no focal abnormalities, including the MRI. In the focal MRI group, reduced verbal automaticity was associated with cerebellar abnormalities, whereas reduced automaticity on motor or motor and verbal tasks was associated with white matter abnormalities. Reduced automaticity of retrieval and slow timed motor performance are highly associated with MRI findings. [1]

COMMENTARY. Previous studies of neurological and MRI profiles of children with cognitive disorders concerned children with developmental language impairment (DLI)[2], and children with memory and learning outcomes 5 years after traumatic brain injury [3]. Investigators from the San Diego School of Medicine, La Jolla, CA, reported 12 (34%) of 35 children aged 5 to 14 years with DLI had abnormalities on the MRI, while only one of 27 control children had abnormal scans [2]. Abnormal MRI findings included ventricular enlargement in 5, central volume loss in 3, and white matter abnormalities in 4. Children with DLI were significantly delayed in motor milestones, especially walking, in addition to speech. Neurological examination abnormalities included synkinesis, fine motor impairments, and hyperreflexia. Children with DLI may need more comprehensive intervention than language therapy alone.

Investigators from the University of Melbourne, Australia, examined the effects of injury severity on long-term memory in 55 children who sustained traumatic brain injury 5 years earlier and compared with 17 healthy controls. Injury severity affected complex memory, with no significant effects on working memory. Diffuse axonal injury predicted outcome on complex memory tasks, but focal cortical damage was not predictive of working or complex memory [3]. The detection of focal brain abnormalities on MRI in children with learning disorders is dependent on the use of higher-resolution scans; the percentage of patients who exhibited brain lesions on MRI when using a 3.0T machine was 60% compared to 32% on a 1.5T machine [1].
